# Acute effects of cluster vs. traditional sets on performance and perceptual responses during upper- and lower-limb power-oriented resistance exercises in older adults

**DOI:** 10.1007/s40520-026-03324-4

**Published:** 2026-01-21

**Authors:** Boliang Wang, Mark Halaki, Derek L. Tran, Timothy B. Davies, Kimberley L. Way, Jonathan Tran, Guy C. Wilson, Glen M. Davis, Maria A. Fiatarone Singh, Daniel A. Hackett

**Affiliations:** 1 Discipline of Exercise and Sport Science, School of Health Sciences, Faculty of Medicine and Health, The University of Sydney, Camperdown, NSW Australia; 2 Musculoskeletal Research Hub, Charles Perkins Centre, The University of Sydney, Camperdown NSW, Australia; 3 Sydney Medical School, Faculty of Medicine and Health, The University of Sydney, Camperdown, NSW Australia; 4 Department of Cardiology, Royal Prince Alfred Hospital, Camperdown, NSW Australia; 5 Institute for Physical Activity and Nutrition, School of Exercise and Nutrition Sciences, Deakin University, Geelong, VIC Australia; 6 Exercise Physiology and Cardiovascular Health Lab, University of Ottawa Heart Institute, Ottawa, ON Canada; 7 Baker Heart and Diabetes Institute, Melbourne, VIC Australia; 8 Hinda and Arthur Marcus Institute for Aging Research, Hebrew SeniorLife, Boston, MA USA

**Keywords:** Resistance training, Muscle fatigue, Ageing, Motor performance, Physical function

## Abstract

**Background:**

Power training is critical for maintaining muscle function and independence in older adults, but excessive fatigue during traditional sets (TRAD) can reduce effectiveness and adherence. Cluster sets (CS) may help counteract these issues; however, most evidence comes from athletes, and acute responses to CS versus TRAD across exercises in older adults remain poorly understood, limiting guidance for optimal prescription.

**Methods:**

Thirty apparently healthy, resistance-trained older adults (19 males, 11 females; 69.3 ± 6.6 years) performed chest press (CP) and leg press (LP) at 70% one-repetition maximum (1RM) at maximal concentric velocity. Participants performed CS (4 × (2 × 5), 30s intra-set rest, 150s between sets; 570s total rest) and TRAD (4 × 10, 180s rest between sets; 540s total rest) on separate occasions in randomized order. Mean concentric velocity (MCV), velocity loss (VL), rating of perceived exertion (RPE), and estimated repetitions to failure (ERF) were measured.

**Results:**

MCV was higher in CS than TRAD for CP (*p* < 0.001) and LP (*p* = 0.005). VL was lower in CS than TRAD for CP (*p* < 0.001) and LP (*p* = 0.003), although CP exceeded 30% VL in both conditions, whereas LP remained below 20% VL. No differences were observed in RPE, whereas ERF was higher in CS for CP (*p* = 0.015) and LP (*p* = 0.045).

**Conclusion:**

CS maintained better exercise performance in older adults, accompanied by perception that they could perform more repetitions compared to TRAD. However, the CS implemented did not significantly reduce perceived exertion.

**Trials Registration:**

This study was registered with the Australian New Zealand Clinical Trials Registry (ANZCTR) under the identifier: ACTRN12622001573741.

## Introduction

Ageing is associated with a decline in muscle function, particularly in muscle strength and power, which are critical determinants of functional independence in older adults [1]. Power training, a form of resistance training that involves performing concentric muscle actions at maximal volitional velocity, is widely recommended to counteract age-related declines in strength and power [1, 2]. To optimize improvements in both parameters simultaneously, recent exercise guidelines for older adults have refined power training recommendations by increasing the prescribed loading range from 40–60% to 60–80% of one-repetition maximum (1RM) [2]. Another notable change in the power training recommendations is that, for the first time, cluster sets (CS) were introduced as an alternative to traditional sets (TRAD) to help mitigate excessive fatigue during training [2].

TRAD is the most commonly used set configuration, involving the consecutive completion of repetitions until the target number per set is reached [3]. A major challenge with TRAD is that, when external load is not adjusted to account for accumulating fatigue, performing 6–10 continuous repetitions per set at approximately 60–80% of 1RM across multiple sets can increase fatigue and bring individuals closer to momentary failure, depending on exercise selection and recovery between sets [4]. Approaching momentary failure may trigger the Valsalva maneuver [5], as well as increase the risk of movement compensations [6]. These responses can result in exaggerated blood pressure elevations and raise the risk of musculoskeletal injury, both of which are particularly concerning in ageing populations [2]. Furthermore, accelerated neuromuscular fatigue is often accompanied by increased perceived exertion [4], which may reduce exercise enjoyment and adherence [7]. Considering both enjoyment and safety are critical considerations for older adults engaging in resistance exercises [2], TRAD may not represent the most suitable set configuration for this population in the context of power training.

On the other hand, CS is an increasingly popular resistance training configuration that was initially popularized among athletic populations and is now being recommended for older adults and clinical populations [8, 9]. Growing evidence supports its effectiveness in better maintaining exercise performance while attenuating muscle fatigue and perceived exertion during power-oriented resistance training in athletes [8, 9]. These benefits may have long-term importance for promoting muscle power development, as well as providing a strategy for load management and injury risk reduction between competitive events [9]. Extending findings from athletes, some authors [10, 11] have suggested that CS may be particularly beneficial for older adults by enhancing safety and facilitating exercise implementation. Since age-related muscle weakness and fatigue can limit exercise capacity and tolerance, CS may help improve training tolerance while minimizing the challenges and risks associated with TRAD [10]. While CS has been incorporated into recent exercise guidelines for older adults, to the best of our knowledge, there is currently only one empirical study that has directly examined the acute effects of CS compared to TRAD in this population [12].

Dello Iacono and colleagues [12] compared various CS structures - varying the number of repetitions per cluster and the rest intervals between clusters - with TRAD during back squats performed at maximal concentric velocity in older males. Using individualized loads that elicited maximal power output, they found that CS protocols were more effective than TRAD in maintaining power output and reducing perceived exertion [12]. While this study [12] provides valuable direct evidence on the benefits of CS in older adults, its generalizability is limited by the inclusion of only male participants and the exclusive use of barbell back squats [13]. It has been suggested that the advantages of CS may only emerge when sets are performed sufficiently close to momentary failure [9]. Upper-body exercises typically reach momentary failure with fewer repetitions than lower-body exercises when relative loads are matched [14]; therefore, prescribing a fixed number of repetitions may result in different levels of relative effort and fatigue across exercise modalities [13, 15]. Consequently, it remains unclear whether the benefits of CS extend similarly across these exercise modalities. Furthermore, practical and increasingly popular measures such as velocity loss (VL), a reliable objective indicator of neuromuscular fatigue [16], and estimated repetitions to failure (ERF), a common surrogate for proximity to momentary failure [17], are commonly used in younger populations and have gradually been introduced in studies involving older adults [18, 19]. Incorporating VL and ERF may provide novel insight into acute responses to CS versus TRAD, potentially informing future use of these tools in prescribing power training for older adults.

Given the growing interest in utilizing CS for power training among older adults [2, 10], our study examined the acute performance and perceptual responses of older adults to CS and TRAD during upper- and lower-body power-oriented resistance exercises using pneumatic devices. Based on evidence from younger populations and a prior study in older adults [9, 12], we hypothesized that CS would better maintain performance and result in different perceptual responses compared with TRAD.

## Materials and methods

### Experimental approach

**Fig. 1:**
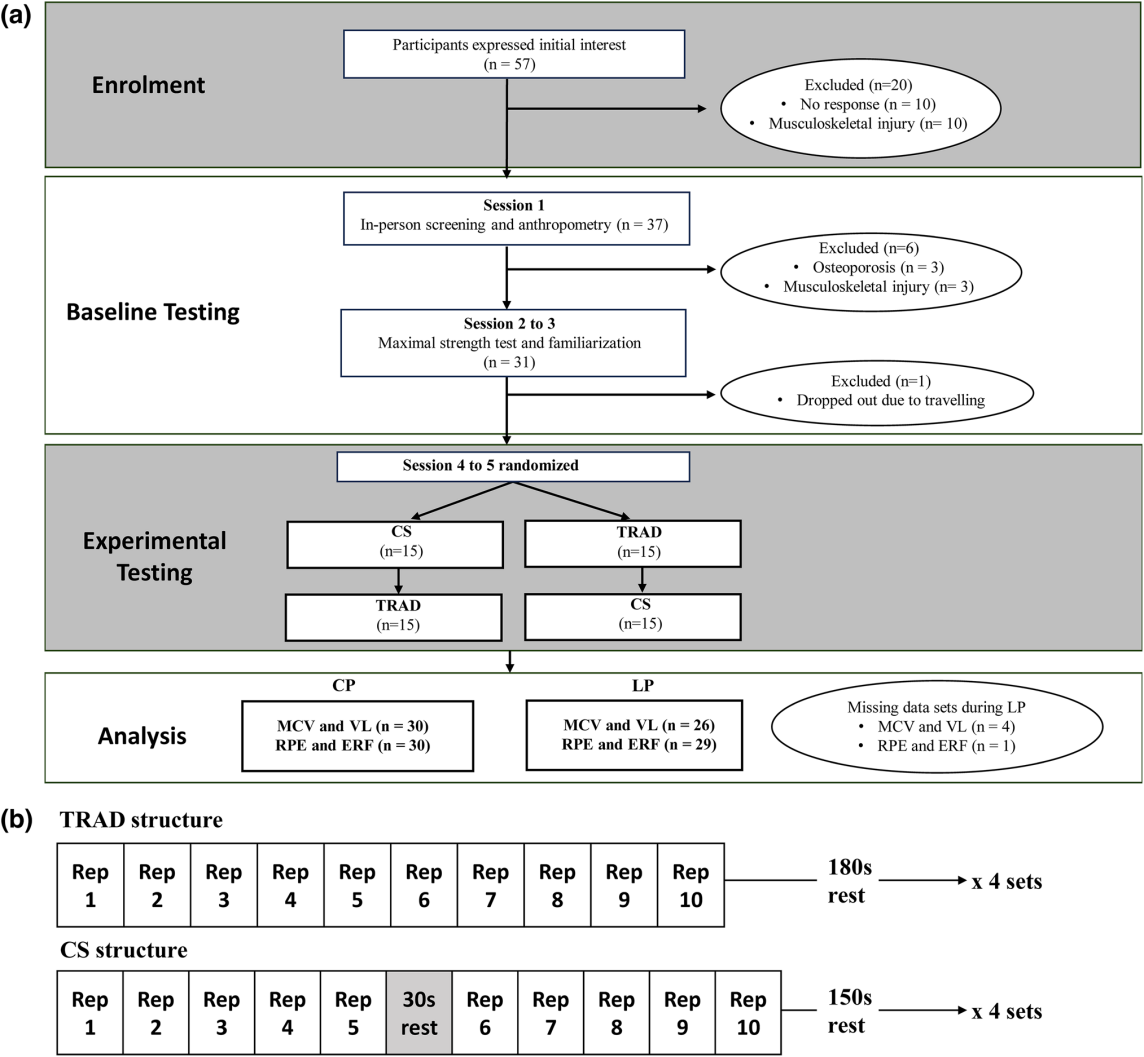
(**a**) Participant flow diagram showing participant flow throughout the study; (**b**) the exercise and set configurations used during experimental testing sessions. CS, cluster sets; TRAD, traditional sets; MCV, mean concentric velocity; VL, velocity loss; RPE, rating of perceived exertion; ERF, estimated repetitions to failure; CP, chest press; LP, leg press; 1RM, one-repetition maximum; Rep, repetition

This study employed a randomized cross-over design to compare exercise performance and perceptual responses between CS and TRAD. The study spanned over a period of 3–6 weeks, during which participants attended five sessions. Baseline assessments during the first three visits included anthropometric measurements (height and body mass) and whole-body composition assessment using dual-energy X-ray absorptiometry (DEXA), followed by two 1RM tests for the chest press (CP) and the leg press (LP) using Keiser A420 pneumatic resistance equipment (Keiser Corporation, Fresno, CA, USA). Pneumatic resistance machines are commonly used in both research and practical settings for older adults [20–22]. The highest 1RM value was used for subsequent experimental sessions. Computer-generated concealed allocation determined whether participants completed CS or TRAD first. In the two experimental sessions, participants performed four sets of 10 repetitions at 70% 1RM for the CP and the LP (in this sequence) using CS or TRAD protocols. This loading intensity was selected to represent a power-oriented resistance exercise load commonly used in older adults [2], while allowing standardisation across participants to isolate the effects of set configuration. Performance outcomes included mean concentric velocity (MCV), defined as the average velocity across the entire concentric phase of each repetition, recorded via the pneumatic equipment’s embedded chip technology. From these data, VL was calculated within each set. Perceptual outcomes included rating of perceived exertion (RPE) and ERF, reported immediately after each set. Session order was randomized using block randomisation (www.random.org). The study flow diagram and participant progression are shown in Fig. 1a, and Fig. 1b illustrates the set configurations and exercise sequence used in the experimental sessions.

The two experimental sessions were scheduled 5–10 days apart to allow for adequate recovery and reduce the effects of any residual fatigue. To control for variations in performance between sessions due to time of day, sessions were held at the same time (± 1 h) for each participant. Participants were instructed to avoid consuming caffeine or pre-workout supplements for 2–3 h before the sessions, refrain from eating within 1 h prior, and abstain from moderate to high-intensity exercise for 48 h before both the 1RM testing and experimental sessions. Additionally, participants were instructed to maintain consistent sleep and dietary habits on the day prior to each session and to record these details for review by research staff upon arrival at the University of Sydney laboratory. Both experimental sessions were conducted individually by the same university-trained exercise physiologist for each participant. 

### Participants

Participants were recruited through advertisement flyers distributed in local gyms and were tested between December 2023 and October 2024 in Sydney, Australia. Eligibility criteria required participants to be 60 years or older and to have a minimum of one year of prior resistance training experience, including familiarity with both the CP and LP exercises. Additionally, participants needed to have trained at least twice a week in the six weeks preceding the study. These criteria were designed to ensure participants could consistently perform high-velocity resistance exercises, minimizing variability due to unfamiliarity with the movement patterns. Participants were excluded if they self-reported or were identified as having any of the following conditions: recent or current musculoskeletal injuries (i.e., fractures or rotator cuff injury within the past year), osteoporosis, acute or terminal illnesses, unstable cardiovascular or respiratory conditions, hernia, or morbid obesity (BMI > 40).

A total of 57 older adults initially expressed interest in participating in the study, 48 completed the online screening, and 38 attended the initial on-site screening. Ultimately, 31 recreationally trained individuals aged 60 to 81 years met the eligibility criteria and were enrolled in the study. One participant withdrew after the second visit due to personal reasons, resulting in 30 participants completing all study sessions (Fig. 1a). The physical characteristics of participants who completed the study are presented in Table 1.

**Table 1:** Physical characteristics of participants

	Male (N = 19)	Female (N = 11)	Total (N = 30)
Age (y)	70.8 ± 6.1	66.7 ± 6.8	69.3 ± 6.6
Height (cm)	174.1 ± 7.9	160.2 ± 6.6 *	169.0 ± 10.0
Body mass (kg)	76.2 ± 11.3	61.9 ± 10.9 *	71.0 ± 13.0
BMI (kg/m²)	25.1 ± 2.6	24.1 ± 4.0	24.7 ± 3.1
Body fat (%)	26.8 ± 7.8	35.7 ± 10.0	30.0 ± 9.6
Training frequency (days/week)	3.2 ± 1.0	2.6 ± 0.6	3.0 ± 0.9
Training experience (years)	11.4 ± 7.8	12.2 ± 6.6	11.7 ± 7.3
Appendicular lean mass (kg)	23.8 ± 2.9	17.6 ± 2.7 *	21.5 ± 4.1
CP 1RM (N)	419.5 ± 90.3	232.3 ± 52.7 *	350.8 ± 120.2
Relative CP strength (N/Kg)	5.6 ± 1.3	3.8 ± 0.9 *	4.9 ± 1.5
LP 1RM (N)	2174.7 ± 390.2	1560.9 ± 290.1 *	1949.7 ± 462.7
Relative LP strength (N/Kg)	28.9 ± 5.6	25.5 ± 3.6	27.7 ± 5.1

BMI = body mass index; CP = chest press; LP = leg press; 1RM = 1-repetition maximum.

Relative CP and LP strength were normalised to kilograms of body mass.

Data were presented as mean ± standard deviation (SD).

Statistical significance was set at p = 0.004 (0.05/12).

* Significant difference between males and females.

This study was carried out in accordance with the Declaration of Helsinki. All procedures were approved by the University of Sydney Human Research Ethics Committee (Project No.: 2022/324), and written informed consent was obtained from each participant before their involvement in the study.

### Procedures

#### Anthropometry, body composition, and bone density

Upon arrival at the laboratory, participants’ height and body mass were measured to the nearest 0.1 cm and 0.1 kg, respectively. Body composition and bone density were assessed using a whole-body DEXA scanner (Lunar Prodigy, GE Medical Systems, Madison, WI, USA) following specific procedures reported in our previous study [23]. Total lean mass and bone mineral density were analyzed using the in-built software (version 13.60.033; enCORE 2011, GE Healthcare, Madison, WI).

#### 1RM testing and familiarization

The 1RM test was conducted first for the CP, followed by the LP, with a 30-minute rest between exercises. Both exercises began with a concentric muscle action followed by an eccentric muscle action. During the CP, seat positions were adjusted to ensure the elbows remained at chest level, minimizing excessive extension and reducing shoulder strain. For the LP, participants were seated in a slightly reclined position with knees bent at approximately 90 degrees flexion, measured using a goniometer. Adjustments were made for individuals with restricted hip mobility to maintain a stable and comfortable posture while minimizing lower back strain. Participants crossed their arms over their chest to limit upper-body involvement, and a Velcro strap secured the torso. Key positioning details, including seat adjustments, handle placements (CP only), and foot placements (LP only), were recorded and replicated in subsequent trials to ensure consistency.

1RM testing was performed using a protocol consistent with our previous work [23]. Briefly, participants completed a standardized warm-up consisting of one set of five repetitions at ~ 50% of perceived 1RM, followed by one to two sets of two to three repetitions at ~ 60–80% of 1RM. Subsequent 1RM attempts involved single repetitions with progressive load increases of ~ 5–10%, interspersed with 3–5 minute rest periods. This process continued until the participant was unable to complete a repetition with proper technique, with 1RM defined as the heaviest successfully completed load.

To ensure consistent execution during the experimental phase, participants completed familiarisation with both the CP and the LP protocols at the end of the third visit. After a 10-20 minute rest following the second 1RM test, they practiced both CS and TRAD protocols using 70% 1RM for one to two sets each, with matched volume across protocols. Participants read the instructions and practiced using both the OMNI resistance exercise scale [24] and ERF scale [25] after each set during this familiarisation session. All participants demonstrated the ability to perform the exercises with the required technique and within the required range of motion before proceeding to subsequent sessions.

#### Experimental sessions

Each experimental session began with participants performing the CP followed by the LP, with a 30-minute rest period between exercises to ensure adequate recovery. Before each exercise, participants completed a standardized warm-up, involving 10 repetitions of the corresponding exercise at 40% 1RM, followed by 1 minute of rest, and then 8 repetitions at 60% 1RM. After 3 minutes of rest, participants performed the designated exercise at 70% 1RM for 4 sets using either the TRAD protocol (10 repetitions each set with a 180s inter-set rest) or the CS protocol (2 clusters of 5 repetitions each set with a 30s intra-set rest and a 150s inter-set rest). The total rest duration for the TRAD and the CS was 540s and 570s, respectively, with the additional 30s in the CS due to the final intra-set rest that does not occur in the TRAD. Each set was designed to include 10 repetitions; however, if the participant could no longer achieve the required range of motion, as assessed by the research staff, that set was prematurely terminated. The number of uncompleted repetitions was recorded.

To promote power output, participants were explicitly instructed to perform the concentric phase of each repetition as fast and explosively as possible, with the verbal cue ‘push’ provided to reinforce this intent. During the eccentric phase, the verbal cue ‘one-two’ was used to guide a controlled two-second lowering of the load. To ensure consistency and reduce variability due to inter-repetition rest, participants maintained a continuous tempo and were instructed to stop the eccentric muscle action approximately 2 cm from the starting position to preserve the stretch-shortening cycle and prevent mechanical ‘bouncing’. For the CP, a custom-built LED display linked to a string potentiometer illuminated as the arms neared the target distance. For the LP, a 2 cm-wide marker placed along the movement path served as a visual cue to standardize range of motion.

#### Mean concentric velocity (MCV) and velocity loss (VL)

During each repetition, the Keiser A420 equipment recorded MCV separately for the left (L) and right (R) sides. The validity of pneumatic resistance equipment for measuring strength, velocity, and power has been established elsewhere [26]. The MCV in each repetition was calculated as the average of the values from the L and R sides. The VL during each set was calculated using the formula: VL = 100 x ($\:{MCV}_{best}-{MCV}_{last})$/ $\:{MCV}_{best}$ [27], where $\:{MCV}_{best}$ represented the highest MCV within each set, and $\:{MCV}_{last}$ represents the MCV of the final repetition within the set; when the final repetition was not completed, $\:{MCV}_{last}$ was obtained via imputation as described in the following section. The highest MCV within each set was used to represent maximal concentric performance, consistent with prior recommendations in younger adults [28] and recent evidence showing that, in machine-based exercises commonly used by older adults, peak MCV does not always occur in the first repetition, likely due to limited stretch-shortening cycle contribution and initiation from a purely concentric phase in the CP and LP [29].

#### Rating of perceived exertion (RPE) and estimated repetitions to failure (ERF)

At the conclusion of each set, participants first rated their RPE using the OMNI resistance exercise scale, ranging from 0 (‘extremely easy’) to 10 (‘extremely hard’) [24]. They then indicated their ERF by specifying the number of additional repetitions they believed could still perform before reaching failure, from ‘10 or more’ to ‘0’ [25]. Both scales were made visible to participants immediately following each set.

### Statistical analyses

Data were expressed as mean ± standard deviation (SD). Normality was evaluated using the Shapiro-Wilk test, and further assessments of skewness and kurtosis confirmed that the raw data for all outcome variables were normally distributed.

Sample size estimation was informed by previous acute work comparing CS and TRAD in older adults that reported a large between-condition effect (Hedges’ g = 1.12) [12]. Based on this effect size, 14 participants were estimated to provide ~ 80% power at α = 0.05 (G*Power 3.1.9.5). Allowing for ~ 20% attrition, a recruitment target of 18 participants was set. Recruitment continued beyond this target to improve the precision of estimates across exercises, resulting in a final sample of 30 participants.

The CP and the LP exercises were analyzed separately. A three-factor repeated measures analysis of variance (ANOVA) was performed for MCV, with condition (CS vs. TRAD), sets (4 sets), and repetitions (10 repetitions) as within-participant factors. Additionally, two-factor repeated measures ANOVAs were conducted for VL, RPE, and ERF, with condition and sets as within-participant factors. Sphericity was assessed using Mauchly’s test for repeated-measures effects with more than two levels, and Greenhouse–Geisser corrections were applied when the assumption of sphericity was violated. Significant differences were further examined using Bonferroni post-hoc tests. Missing CP data were addressed via an imputation method based on the average slope across participants, incorporating participant-specific intercepts to preserve individual trends. To assess the robustness of the findings, a sensitivity analysis was conducted for MCV and VL during the CP using a complete-case approach, which yielded comparable results. Effect sizes (ES) were calculated using partial eta-squared ($\:{{\upeta\:}}_{p}^{2}$) values. The ES were categorized as small ($\:{{\upeta\:}}_{p}^{2}$ = 0.01 to < 0.06), medium ($\:{{\upeta\:}}_{p}^{2}$ = 0.06 to < 0.14), or large ($\:{{\upeta\:}}_{p}^{2}$ ≥ 0.14) [30].

Baseline sex differences were assessed with independent samples t-tests, applying a Bonferroni-adjusted significance level of 0.004 (0.05/12) for multiple comparisons. All other analyses used a significance threshold of *p* < 0.05. The reliability of the 1RM test was assessed with a two-way mixed model intraclass correlation coefficient [ICC (3,1)], with results indicating excellent reliability (ICC = 0.99 for both the CP and the LP). Statistical analyses were conducted using Jamovi v.2.3.28 (Jamovi, Sydney, Australia).

## Results

### Missing data and adverse events

For the CP, three females and two males in the TRAD condition and one male in the CS condition did not complete all 40 repetitions, with most missing values occurring in sets 3 and/or 4. These accounted for 20 out of 2,400 total repetitions (0.83%) for MCV in the CP. All CP outcome analyses were conducted with a sample size of *n* = 30 after data imputation, as previously described. For the LP, performance data (MCV and VL) were missing for four LP sessions: three due to technical failure resulting in unsaved data (two CS sessions and one TRAD session) and one TRAD session discontinued when a male participant experienced a minor hamstring cramp. Symptoms subsided after stopping, but the session was not repeated. Perceptual data (RPE and ERF) were available for all sessions except the discontinued TRAD session. Consequently, LP performance data were analyzed with *n* = 26, and perceptual outcomes with *n* = 29. No other adverse events were reported.

### Mean concentric velocity (MCV)

For the CP, a significant condition × repetitions interaction effect (*p* < 0.001, partial $\:{{\upeta\:}}_{p}^{2}$ = 0.456) was found, and post-hoc analyses revealed that MCV during repetitions 7–10 was greater for CS than TRAD. Significant main effects were observed for condition (*p* < 0.001, partial $\:{{\upeta\:}}_{p}^{2}$ = 0.391), sets (*p* < 0.001, partial $\:{{\upeta\:}}_{p}^{2}$ = 0.543), and repetitions (*p* < 0.001, partial $\:{{\upeta\:}}_{p}^{2}$ = 0.852). Overall, MCV was higher for CS than TRAD (0.60 ± 0.15 vs. 0.55 ± 0.15 m·s^− 1^, mean difference = 0.05 m·s^−1^, 95% CI: 0.026 to 0.073), with a progressive decline across sets.

**Fig. 2:**
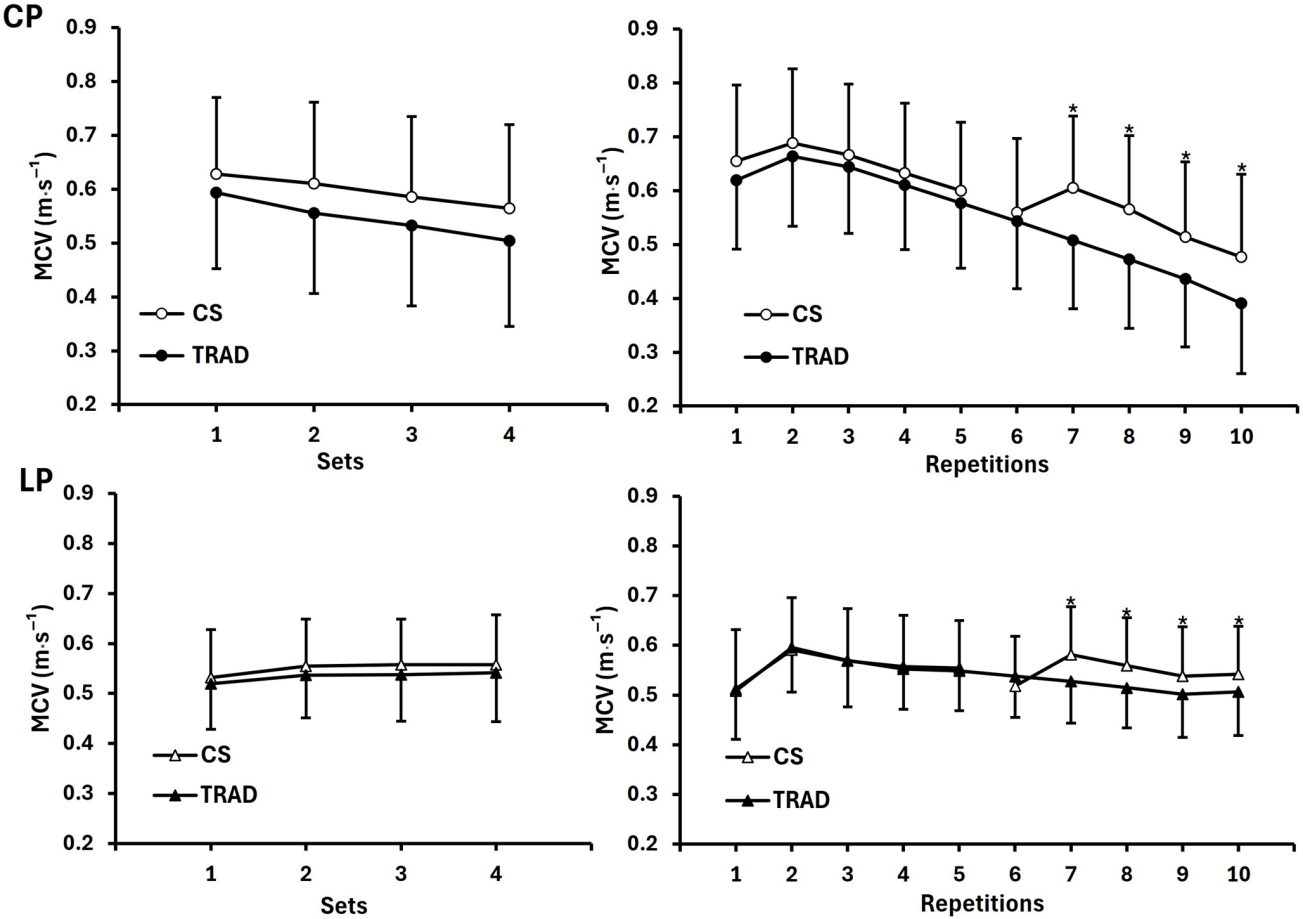
MCV comparisons between CS and TRAD set configurations during the CP (*n* = 30) and LP (*n* = 26). Each panel includes two sub-graphs: the left sub-graphs show MCV averaged across all repetitions within each set (sets 1–4), and the right sub-graphs display MCV averaged across all sets for each repetition (repetitions 1–10). *MCV in the CS was significantly different from the TRAD (*p* < 0.05). MCV, mean concentric velocity; CS, cluster sets; TRAD, traditional sets; CP, chest press; LP, leg press

For the LP, a significant condition × repetitions interaction (*p* < 0.001, partial $\:{{\upeta\:}}_{p}^{2}$ = 0.347) was noted, and post-hoc analyses revealed MCV was greater for CS than TRAD during repetitions 7–10. Significant main effects were also found for condition (*p* = 0.005, partial $\:{{\upeta\:}}_{p}^{2}$ = 0.275), sets (*p* < 0.001, partial $\:{{\upeta\:}}_{p}^{2}$ = 0.355), and repetitions (*p* < 0.001, partial $\:{{\upeta\:}}_{p}^{2}$ = 0.445). Overall, MCV was higher for CS than TRAD (0.55 ± 0.10 vs. 0.53 ± 0.09 m·s^− 1^; mean difference = 0.02 m·s^−1^, 95% CI: 0.006 to 0.028), with no significant changes observed across sets. MCV averaged across all repetitions within each set (1–4) and averaged across all sets for each repetition (1–10) during the CP and the LP are shown in Fig. 2.

### Velocity loss (VL)

For the CP, significant main effects for VL were found in condition (*p* < 0.001, partial $\:{{\upeta\:}}_{p}^{2}$ = 0.517) and sets (*p* < 0.001, $\:\mathrm{p}\mathrm{a}\mathrm{r}\mathrm{t}\mathrm{i}\mathrm{a}\mathrm{l}\:{{\upeta\:}}_{p}^{2}$ = 0.542). Post-hoc testing revealed that VL was lower for CS than TRAD (33.5 ± 13.5% vs. 43.4 ± 13.5%; mean difference = − 9.9%, 95% CI: −13.5 to − 6.3). Additionally, there was a gradual increase in VL between each set, except between sets 2 and 3.

**Fig. 3:**
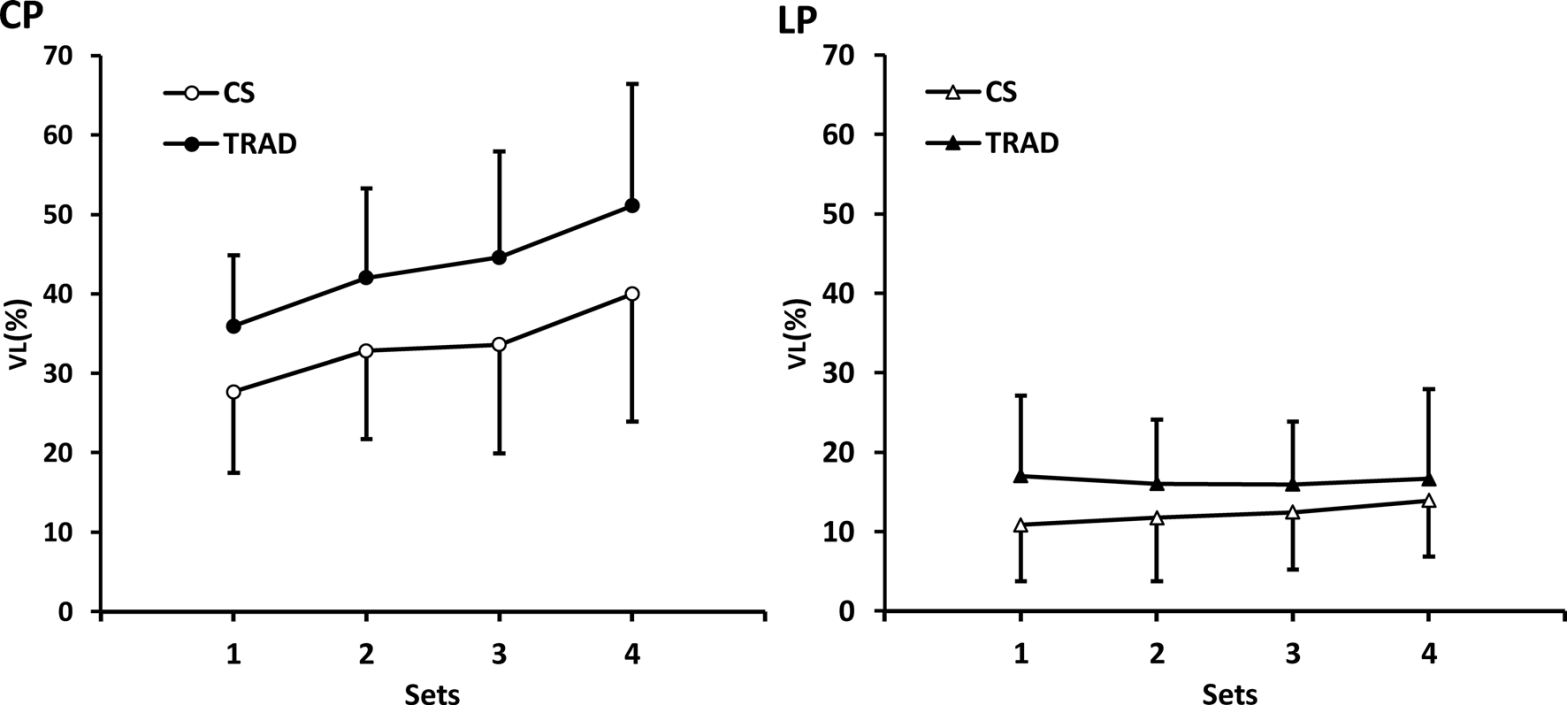
VL comparisons between CS and TRAD set configurations during chest press (*n* = 30) and leg press (*n* = 26). Data represent mean ± SD. VL, velocity loss; CS, cluster sets; TRAD, traditional sets; CP, chest press; LP, leg press

For the LP, there was a significant main effect for condition (*p* = 0.003, partial $\:{{\upeta\:}}_{p}^{2}$ = 0.301) on VL, with CS lower than TRAD (12.2 ± 7.3% vs. 16.4 ± 9.3%; mean difference = − 4.2%, 95% CI: −6.8 to − 1.6). No other significant main effects or interactions with conditions were observed. The complete figure illustrating VL during each set in the CP and the LP is shown in Fig. 3 .

### Rating of perceived exertion (RPE) and estimated repetitions to failure (ERF)

For the CP, no significant main effect of condition was observed for RPE, with similar values between CS and TRAD (7.7 ± 1.2 vs. 8.0 ± 1.4; mean difference = − 0.29, 95% CI: −0.65 to 0.07). There was a significant main effect of sets on RPE (*p* < 0.001, partial $\:{{\upeta\:}}_{p}^{2}$ = 0.740), with post-hoc analysis indicating a progressive increase across sets. For ERF, significant main effects were found for condition (*p* = 0.015, partial $\:{{\upeta\:}}_{p}^{2}$ = 0.187) and sets (*p* < 0.001, partial $\:{{\upeta\:}}_{p}^{2}$ = 0.354). Post-hoc analysis revealed that ERF was higher in the CS than TRAD (2.88 ± 1.66 vs. 2.11 ± 1.80; mean difference = 0.77, 95% CI: 0.16 to 1.38), with ERF showing a progressive decline across sets. No other significant main effects or interactions involving condition were observed.

For the LP, no significant main effect of condition was observed for RPE, with similar values between CS and TRAD (7.4 ± 1.1 vs. 7.7 ± 1.2; mean difference = − 0.32, 95% CI: −0.68 to 0.04). A significant main effect of sets on RPE was found (*p* < 0.001, partial $\:{{\upeta\:}}_{p}^{2}$ = 0.384), with post-hoc analysis indicating a progressive increase across sets, except between Set 2 and Set 3, where no significant difference was observed. For ERF, a significant main effect of condition was found (*p* = 0.045, partial $\:{{\upeta\:}}_{p}^{2}$ = 0.136), with post-hoc analysis revealing higher ERF in the CS than TRAD (3.8 ± 1.9 vs. 3.2 ± 1.5; mean difference = 0.57, 95% CI: 0.01 to 1.13). There was also a significant main effect of sets on ERF (*p* < 0.001, partial $\:{{\upeta\:}}_{p}^{2}$ = 0.271), with Set 1 showing higher ERF than Sets 2–4, while no significant differences were observed among Sets 2, 3, and 4. No other significant main effects or interactions with condition were observed. Figure 3 compares RPE and ERF between CS and TRAD during the CP and the LP.

**Fig. 4:**
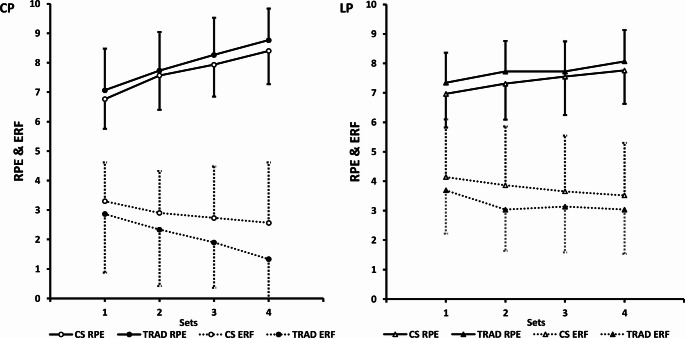
RPE and ERF comparisons between CS and TRAD set configurations during CP (*n* = 30) and LP (*n* = 29). Data represent mean ± SD. RPE, rating of perceived exertion; ERF, estimated repetitions to failure; CS, cluster sets; TRAD, traditional sets; CP, chest press; LP, leg press

## Discussion

Although there is growing interest in the benefits of CS for older adults [2, 10], much of the existing evidence is based on studies with younger populations [8, 9]. This study investigated the acute performance and perceptual effects of CS compared to TRAD, across both upper- and lower-body power-oriented resistance exercises in older adults. The main findings revealed that: (a) CS maintained higher absolute MCV in both the CP and the LP, particularly during the latter half of each set (repetitions 7–10); (b) VL, a marker of neuromuscular fatigue, was significantly lower in CS compared to TRAD for both exercises. However, descriptive data showed that VL during the CP exceeded the 20–25% range commonly recommended in younger cohorts [28], whereas average VL during the LP was lower than this range; and (c) no significant differences in RPE were found between CS and TRAD, despite ERF being significantly higher in the CS condition for both exercises.

Overall, CS resulted in higher MCV than TRAD, with significant effects observed only in the second cluster of repetitions. This pattern is consistent with the potential role of phosphocreatine replenishment during the additional intra-set rest provided by CS [8], which has been proposed to support ATP resynthesis and the maintenance of power output [4]. Maintaining higher movement velocity aligns with the principle of training specificity [31], which suggests that training adaptations are most effective when they closely match the performance demands. Training at higher actual MCV may also offer additional benefits for trained individuals aiming to further enhance power performance [31]. Additionally, CS significantly reduced VL compared to TRAD. When repetitions are executed at maximal volitional velocity, VL provides a practical, non-invasive method to quantify muscle fatigue [16], which can, in turn, influence long-term training adaptations. Evidence from younger populations has indicated that power training with an approximate 20–25% VL threshold yields greater improvements in muscle hypertrophy and strength adaptations compared to minimal VL thresholds (i.e., 0%) [28]. This moderate VL threshold also preserves fast-twitch fiber recruitment, which is essential for power development, as opposed to higher VL thresholds (~40%) [32]. Therefore, the attenuated reduction in VL observed with CS may be especially important for older adults aiming to optimize power output, which is critical for maintaining functional independence and performing daily tasks.

Despite the potential practical implications of reduced VL with CS, it should be noted that VL thresholds have not yet been clearly established for older adults, although emerging evidence has begun to explore their relevance. A recent study [18] reported that in older men using velocity-based training at loading intensities of 40–65% 1RM, both 10% and 20% VL thresholds improved LP strength and velocity, CP velocity, and sit-to-stand performance, while the 20% threshold additionally improved handgrip strength and walking speed [18]. However, it remains unclear whether higher VL thresholds (i.e., ~40%) would produce similar, more beneficial, or adverse outcomes in older populations. Furthermore, exercise selection and loading also substantially influence the muscle effort required to complete a given number of repetitions or to reach a specific VL threshold [28, 33]. This, in turn, affects neuromuscular fatigue and may lead to differing long-term adaptations [28, 33].

In the current study, VL during the LP was relatively low for both CS (12.2%) and TRAD (16.4%) and may already be sufficient to promote power adaptations based on VL thresholds previously proposed in younger populations. Therefore, whether CS offers additional long-term benefits for this exercise remains uncertain. In contrast, the CP exhibited higher VL values of 33.5% for CS and 43.4% for TRAD, exceeding VL thresholds commonly proposed in younger populations [28] and suggesting that both protocols may have elicited substantial neuromuscular fatigue. Although no direct physiological measures were analyzed in this study to confirm this, exercise termination occurred only during CP, potentially reflecting greater neuromuscular fatigue demands of this exercise. Collectively, these findings suggest that, at 70% 1RM, the CP may require more frequent intra-set rest intervals to better manage VL in older adults, even though CS attenuated VL compared with TRAD. Future studies should seek to identify optimal VL thresholds across different exercises and specific muscle adaptations of interest (e.g., muscle strength, power) in older adults, which may help inform the design of CS configurations targeting these thresholds. Future work may also explore combining the current CS configuration with alternative strategies to manage VL during upper-body exercises. In particular, active inter-set rest using very low loads (e.g., 5–10% 1RM) and exercise-specific movements has been shown to reduce VL during the bench press in younger adults [34, 35] and therefore warrant investigation in older adults.

Contrary to our hypothesis and evidence from younger populations [9], RPE remained unchanged with CS compared to TRAD in older adults across both exercises. This may reflect limitations in our CS structure, whereby providing intra-set rest only after five consecutive repetitions may be inadequate for older adults. Consistent with this interpretation, Dello Iacono and colleagues [12] demonstrated that older adults reported significantly lower RPE when using CS with more frequent rest intervals (i.e., after every 1–2 repetitions) compared to both TRAD and CS with longer clusters (i.e., 4 repetitions). This suggests older adults may require shorter, more frequent rest periods to manage perceived exertion effectively. It should also be noted that the type of resistance equipment used may influence perceptual responses. Pneumatic resistance machines differ in their mechanical properties from free-weight or isotonic machines and may alter force-velocity characteristics and sensory feedback [36], potentially influencing how effort is perceived during exercise.

While RPE remained similar between set configurations, participants reported higher ERF values during the CS, indicating a perceived greater capacity to complete additional repetitions. Recent work in older adults suggests that ERF may be more sensitive than RPE for detecting neuromuscular fatigue, as RPE can remain relatively stable across sets despite substantial differences in VL [37]. Although this work also indicates that older adults may systematically underestimate ERF [37], the observed elevation in ERF with CS occurred concurrently with the measured reduction in VL relative to TRAD, suggesting ERF may function as a perceptual counterpart to the objective fatigue attenuation demonstrated by VL measurements. Using ERF may be a practical, equipment-free method for clinicians working with older adults to monitor fatigue levels when velocity-tracking technology is unavailable. From a practical perspective, these programming considerations should be individualised, with practitioners integrating objective (MCV, VL) and perceptual (RPE, ERF) markers, where available, to guide adjustments in set configuration based on individual tolerance and training status.

While this study provides novel insights into the application of CS for older adults, several limitations that constrain the findings should be acknowledged. The inclusion of only healthy, resistance-trained older adults limits the generalisability of the findings to untrained individuals or those with existing health conditions. In addition, the uneven distribution and limited number of male and female participants limited the ability to investigate potential sex-related differences in responses to CS versus TRAD; while future adequately powered studies could explore this, prior evidence in younger adults indicates that such sex-related effects are small or inconsistent [38, 39]. Moreover, the benefit of CS seen in the current study might be specific to power training [8], and such training mode may not be suitable for older adults with musculoskeletal limitations who need to avoid high-velocity movements [2]. Consequently, important questions remain to be addressed regarding whether conventional resistance training with controlled movement velocities can benefit from CS. Future research should explore this possibility and expand the scope to include health-related outcomes, such as cardiovascular load and hemodynamic responses, which may reveal additional advantages of CS for older adults.

The current study also focused only on one specific CS configuration with extended repetition clusters. Future research should investigate the effects of varying intra-set rest durations and different cluster repetition schemes on acute training responses. In addition, the order of the CP and LP exercises was not randomised. Although the between-exercise recovery period was standardised to minimise fatigue-related carryover effects, the fixed sequence may still represent a limitation. Finally, while VL is commonly used as a fatigue measure in younger populations, early termination in older adults may be due to perceived discomfort or safety concerns, rather than true neuromuscular fatigue. Therefore, caution is needed when interpreting VL as a standalone marker of fatigue, and complementary assessments like electromyography (EMG) may provide further insights into neuromuscular fatigue in these contexts [40].

Investigating whether the current findings translate into greater long-term neuromuscular adaptations is vital. Existing literature presents conflicting evidence on the long-term effectiveness of CS compared to TRAD interventions in ageing populations, with some studies demonstrating superior functional improvements compared to TRAD [41] while others show no significant differences [42, 43]. These discrepancies likely stem from variations in experimental protocols, particularly in the CS configuration and implementation parameters (i.e., loading intensity and number of sets). The acute responses observed in the current study, including velocity preservation patterns between different exercises, provide a valuable framework for designing more effective longitudinal interventions that account for the unique physiological considerations of older adults.

## Conclusion

There were greater benefits of CS over TRAD in maintaining exercise performance and perception of greater capacity to complete more repetitions after each set compared to TRAD in healthy, trained older adults, while eliciting comparable RPE. However, whether these acute responses will translate into superior long-term muscle adaptations, particularly in older adults new to power training or those with clinical conditions, remains unclear. These aspects warrant further investigation to better inform programme design across diverse ageing populations.

## Data Availability

The datasets generated and/or analyzed during the current study are not publicly available due to ethical considerations but are available from the corresponding author upon reasonable request.
